# Doctors' adherence to guidelines recommendations and glycaemic control in diabetic patients in Quetta, Pakistan: Findings from an observational study

**DOI:** 10.3389/fmed.2022.978345

**Published:** 2022-10-31

**Authors:** Tabassum Saher, Yaser Mohammed Al-Worafi, Muhammad Nouman Iqbal, Abdul Wahid, Qaiser Iqbal, Asad Khan, Muhammad Atif, Nafees Ahmad

**Affiliations:** ^1^Department of Pharmacy Practice, Faculty of Pharmacy and Health Sciences, University of Baluchistan, Quetta, Pakistan; ^2^Department of Clinical Sciences, College of Pharmacy, University of Science and Technology of Fujairah, Fujairah, United Arab Emirates; ^3^Sardarpur Rural Health Centre, Khaniwal, Pakistan; ^4^Department of Pharmaceutics, Faculty of Pharmacy and Health Sciences, University of Baluchistan, Quetta, Pakistan; ^5^Department of Pharmacy Practice, Faculty of Pharmacy, The Islamia University of Bahawalpur, Bahawalpur, Pakistan

**Keywords:** chronic kidney diseases, diabetes mellitus, glycemic control, metformin, polytherapy

## Abstract

**Background:**

Poor control of diabetes mellitus (DM) is partly attributed to doctors' poor adherence to guidelines.

**Objective:**

To evaluate doctors' adherence to pharmacotherapeutic recommendations of DM management guidelines and factors associated with guidelines adherence and glycaemic control.

**Methods:**

This prospective observational study included 30 doctors who were treating DM patients in their private clinics in Quetta, Pakistan. On visit 1, a total of 600 prescriptions written by 30 enrolled doctors (20 patients per doctor) were noted along with patients' sociodemographic and clinical characteristics. American Diabetes Association guidelines was used as a reference. The prescriptions noted were judged for guidelines compliance. Of 600 enrolled patients, 450 patients (15 patients per doctor) were followed for one more visit and included in final analysis. Glycated hemoglobin (HbA1c) level noted one visit 2 was related with the respective prescription on visit 1. Data were analyzed by SPSS (version 23). A *p*-value <0.05 was considered statistically significant.

**Results:**

Patients received a median of two antidiabetic drugs (range: 1–5). A total of 73.1% patients were on polytherapy. Metformin was the most frequently prescribed (88.4%) antidiabetic followed by gliptins (46.2%). A total of 41.6% prescriptions were judged guidelines compliant. In multivariate binary logistic regressions (MVBLR) analysis, chronic kidney disease (CKD) (OR = 0.422) and polytherapy (OR = 0.367) had statistically significant negative associations (*p*-value <0.05) with guidelines' compliant prescriptions. The group of doctors comprised of specialists and consultants wrote significantly (*p*-value = 0.004) high number of guidelines adherent prescriptions (mean rank = 20.25) than the group comprised of medical officers (mean rank = 11.34). On visit 2, only 39.5% patients were on goal glycemic levels. In MVBLR analysis, suffering from dyslipidemia (OR = 0.134) and CKD (OR = 0.111), receiving sulfonylurea (OR = 0.156) and guidelines' compliant prescription (OR = 4.195) were significantly (*p*-value <0 .05) associated with glycemic control.

**Conclusion:**

Although guidelines compliant prescriptions produced better glycemic control, but doctors' adherence to guidelines and glycemic control were poor. Polytherapy and CKD emerged as risk factors for guidelines divergent prescriptions. Dyslipidemia, CKD and reception of sulfonylureas had negative association with glycemic control.

## Introduction

Diabetes mellitus (DM) characterized by hyperglycemia is a chronic, complex metabolic syndrome resulting from either lack of insulin release, insulin resistance or both (1). According to International Diabetes Federation (IDF), it is one of the fastest growing global health emergencies. In 2021, approximately 537 million people were living with DM (2) and 6.5 million deaths in 20–79 years old people were attributed to DM and its complications (2). Despite its high prevalence and associated complications, glycemic control in diabetic patients is poor and ranges from 23.4 to 60% (3–6). In order to achieve the target glycemic levels and prevent and reduce complications of DM, a number of clinical practice guidelines have been published, regularly updated and widely disseminated by different organizations like IDF, the American Diabetes Association (ADA) etc., (7, 8). These guidelines contain recommendations based on high quality evidence obtained from large and rigorously conducted randomized control trials, individual patients' data meta-analyses, systematic reviews and expert opinions (9, 10). Compliance with these guidelines have resulted in optimal glycemic control and preventing micro and macrovascular complications of DM (11–18). Nevertheless, a wide gap is present between guidelines recommendations and actual clinical practices (19–36). Non-pharmacological management contributes to optimal glycemic control, but pharmacotherapy is the most effective and commonly used medical intervention in treating DM. However, majority of the previously conducted studies have evaluated standards of diabetic care processes and targets (20, 29–35), and only few have examined doctors' adherence to pharmacotherapeutic recommendations of guidelines. Studies which have assessed doctors' adherence to pharmacotherapeutic recommendations of guidelines have done it by (i) evaluating anti-hyperglycemic prescription patterns and prescribing trends over time (24–28), (ii) review of patients' medical records (15, 18, 20–23, 36) and (iii) assessing doctors' knowledge about guidelines recommendations (19). These studies have certain worth mentioning limitations like lack of an explicit criterion to define doctor's adherence to pharmacotherapeutic recommendations of guidelines (24–28), use of self-reported practices as a proxy of objective adherence to guidelines (19, 37) which are always subject to bias as doctors may overestimate their adherence to guidelines (38, 39), exclusion of patients with comorbidities, lack of detailed review of patient medical record to note that whether doctors' non-adherence to guidelines was a justifiable one or not (20–22, 24–28, 37).

Unfortunately, Pakistan is one of those three countries (China, India and Pakistan) which not only harbors the largest number of diabetic patients but with estimated comparative prevalence of 33.6% in people of age 20–79 years, it currently ranks first globally. In order to evaluate and improve the standard of care provided to diabetic patients, it is necessary to assess the doctors' adherence to guidelines. Due of scarcity of published information in this regard from Pakistan, and to overcome the abovementioned limitations associated with the previously conducted studies, this study was undertaken with the aim to evaluate the doctors adherence to pharmacotherapeutic recommendations of DM guidelines against an explicit criteria, factors associated with receiving guidelines compliant prescriptions and glycemic control.

## Methods

### Study design, settings, and population

This was an observational study conducted at private clinics in Quetta city from June 1, 2021 to December 31, 2021. After getting a written consent, a total of 30 doctors who were involved in treating diabetic patients were enrolled in the study. As we aimed to examine the prescriptions written by each enrolled doctor to a convenience sample of 15 established diabetic ambulatory patients, thus a total of 600 patients (20 patients were per the enrolled doctor) with a potential dropout rate of 25% were included in the study. Patients who were younger than 18 years, who were pregnant, asked to follow-up in <3 months and did not come for the follow-up visit were excluded from the study.

### Data collection

On the baseline visit, a purpose designed data collection form was used to collect patients' sociodemographic, clinical and laboratory data from their medical records and if needed one-on-one interview with the patient. Diagnosis of DM and other concurrent clinical conditions were based in documentation in the patients' medical record. Multiple concurrent clinical conditions like hypertension, dyslipidemia, chronic kidney diseases (CKD) etc., were noted and reported individually, whereas coronary heart diseases and heart failure were collectively categorized as cardiovascular diseases (CVD). Medicines prescribed were noted by their generic names. Treatment with antidiabetic medications with single active pharmaceutical ingredient (API) was termed as monotherapy, whereas, treatment with more than one API (either in fixed dose combination or two different individual drugs) was termed as multi-therapy. In order to find a reason for doctors' justifiable non-adherence with guidelines recommendations, a detailed review of the patients' medical records was carried out. Contraindications, adverse events and statement about ineffectiveness due to which the guidelines recommended drug is not prescribed, discontinued or changed were noted. A total of 450/600 enrolled patients (15 patients/enrolled doctor) were followed for one more visit. One the follow up visit which on average took 3.5 months, patients were tested for glycated hemoglobin (HbA1c) and results were noted. Based on HbA1c values recommended by ADA guidelines (<7% in patients without any comorbidity and <8% in patients with comorbidity) patients were categorized into those having controlled and uncontrolled HbA1c. The criterion developed on the basis of ADA guidelines (Supplementary Chart 1) was used for assessing the doctors' adherence to pharmacotherapeutic recommendations (7). A prescription was judged as guidelines' compliant when:

i) ADA guidelines preferred first-line agent for the particular condition was prescribed.
ii) ADA guidelines preferred first-line agents with no contraindications to its use were prescribed to the patients with comorbidities.
iii) ADA guidelines preferred first-line agent for a particular condition was not prescribed due to adverse event supposedly caused by the preferred drug, contraindication or ineffectiveness.

Score of “1” and “0” were, guidelines' adherent and non-adherent prescription.

### Data analysis

Statistical Package for Social Sciences (SPSS v 23) was used for analyzing data. In order to find out factors associated with receiving guidelines compliant prescription and uncontrolled HbA1c, those variables which had a *p*-value (<0.20) in univariate analysis were entered into multivariate binary logistic regression analysis (MVBLRA). Furthermore, Mann-Whitney U and Kruskal Wallis tests wherever applicable were to observe difference between doctors' demographics and guidelines adherence scores. A *p*-value <0.05 was considered statistically significant.

## Results

The sociodemographic characteristics of enrolled doctors, and sociodemographic and clinical characteristics of 450 patients included in the final analysis are, respectively given in Tables 1, 2. The mean age of patients was 49.03 ± 11.87 years. Majority of them were males (61.3%), overweight (46.9%), had DM for >5 years (73.6%) and >2 comorbidities (43.1%) with hypertension being the most prevalent comorbidity (78.9%).

**Table 1:** Sociodemographic characteristics of doctors.

**Variables**	**No. (%)**
**Gender**	
Male	28 (93.3)
Female	2 (6.7)
**Age**	
25–35	6 (20)
36–45	17 (56.7)
>46	7 (23.3)
**Designation**	
Medical officer	16 (53.3)
Consultant	8 (26.7)
Specialist	6 (20)
**Practice tenure**	
≤ 5year	3 (10)
>5year	27 (90)

**Table 2:** Sociodemographic and clinical characteristics of patients.

**Variables**	**No. (%)**
**Gender**	
Male	276(61.3)
Female	174 (38.7)
**Age** (years)	
25–30	129 (28.7)
41–55	168 (37.3)
>55	123 (34.0)
**Body mass index** (kg/m^2^)	
Underweight (< 18.5)	8 (1.8)
Normal (18.5–24.9)	69 (15.3)
Overweight (25.0–29.9)	211 (46.9)
Obese (>30.0)	162 (36.0)
**Duration of diabetes mellitus**	
<1 year	11 (2.4)
1–5 year	108 (24.0)
>5 year	331 (73.6)
**Family history of cardiovascular diseases**	295 (65.6)
**Comorbidities**	
No	180 (40.0)
1–2	76 (16.9)
>2	194 (43.1)
**Type of comorbidity**	
Stroke	49 (10.1)
Hypertension	355 (78.9)
Dyslipidemia	245 (54.4)
Cardiovascular disease	114 (25.3)
Heart failure	20 (4.4)
Coronary heart disease	97 (21.6)
Chronic kidney disease Stage-III a (eGFR = 45–59 ml/min/1.73 m^2^) Stage-III a (eGFR = 45–59 ml/min/1.73 m^2^) Stage-4 (eGFR = 15–29 ml/min/1.73 m^2^) Stage-5 (eGFR < 15 ml/min/1.73m^2^) Stage unknown	86 (19.1) 33 17 11 4 21
Others	110 (24.4)

eGFR, estimated glomerular filtration rate; min, minute; ml, milliliter; m, meter.

Patients received a median of 2 antidiabetic drugs (range: 1–5). Only 121 (26.9%) patients were on monotherapy. Among antidiabetics, metformin was the most commonly prescribed drug (88.4%) followed by gliptins (46.2%) (Table 3).

**Table 3:** Prescription of antidiabetics.

**Variables**	**No. (%)**
**Number of antidiabetics prescribed**	
1 2–3 >3	121 (26.9) 124 (49.8) 105 (23.3)
**Antidiabetics prescribed**	
Metformin	398 (88.4)
DDP4 inhibitors	208 (46.2)
Insulin	177 (39.3)
Sulfonylureas	150 (33.3)
SGLT-2 inhibitors	74 (16.4)
GLPRA	9 (2.0)

DPP4, Dipeptidyl peptidase-4; GLPRA, Glucagon-like Peptide-1 receptors agonists; SGLT2, Sodium-glucose cotransporters-2.

Out of 450 prescriptions, 187 (41.6%) were judged guidelines compliant. In multivariate analysis, suffering from CKD (OR = 0.422, *p*-value = 0.012) and polytherapy (OR = 0.367, *p*-value < 0.001) had statistically significant association with receiving guidelines' compliant prescriptions. This model fit was based on non-significant Hosmer Lemeshow (*p*-value = 0.287) and overall percentage 65.3% from classification table (Table 4). On the basis of designation, doctors were divided into two groups, that is, medical officers and others (specialists and consultants). Mann–Whitney *U* test revealed a significant difference (*U* = 45.00, *p*-value = 0.004) between the guidelines adherent practices by two groups. The group comprised of specialists and consultants wrote significantly high number of guidelines adherent prescriptions (mean rank = 20.25) than the group comprised of medical officers group (mean rank = 11.34). However, there was no significant difference between the guidelines adherent practices on the basis of doctors' gender, age, place of graduation and practice duration.

**Table 4:** Factors associated with receiving guidelines compliant prescriptions.

**Variables**	**Guidelines compliant Rx No. (%)**	**Univariate analysis**		**Multivariate analysis**	
		**OR (95%CI)**	***p*** **-value**	**OR (95%CI)**	***p*** **-value**
**Gender**					
Male	113 (40.9)	Referent		-	
Female	74 (42.5)	1.067 (0.727–1.568)	0.739	-	
Old age^*^					
No	145 (44.5)	Referent		Referent	
Yes	42 (33.9)	0.639 (0.415–0.984)	0.042	1.379 (0.767–2.479)	0.284
**Body mass index** (kg/m^2^)					
Underweight (<18.5)	2 (25.0)	Referent			
Normal (18.5–24.9)	32 (46.4)	2.595 (0.489–13.766)	0.263	-	
Overweight (25.0–29.9)	97 (46.0)	2.553 (0.504–12.938)	0.258	-	
Obese (>30.0)	56 (34.6)	1.585 (0.310–8.111)	0.580	-	
**Comorbidities**					
No	86 (47.8)	Referent		Referent	
1–2	35 (46.1)	0.933 (0.545–1.597)	0.801	1.301 (0.729–2.323)	0.374
>2	66 (34.0)	0.564 (0.371–0.855)	0.007	1.500 (0.756–2.974)	0.246
**Hypertension**					
No	43 (45.3)	Referent		-	
Yes	144 (40.0)	0.825 (0.523–1.302)	0.409	-	
**Dyslipidemia**					
No	92 (44.9)	Referent			
Yes	95 (38.0)	0.778 (0.534–1.134)	0.191	-	
**Chronic kidney diseases**					
No	165 (45.3)	Referent		Referent	
Yes	22 (25.6)	0.415 (0.245–0.702)	0.001	0.422 (0.214–0.830)	0.012
**Cardiovascular diseases**					
No	150 (44.6)	Referent		Referent	
Yes	37 (32.5)	0.596 (0.381–0.932)	0.023	0.849 (0.448–1.610)	0.617
**Stroke**					
No	175 (43.6)	Referent		Referent	
Yes	12 (24.5)	0.419 (0.212–0.827)	0.012	0.506 (0.236–1.084)	0.080
**Other diseases**					
No	146 (42.9)	Referent			
Yes	41 (37.3)	0.790 (0.507–1.229)	0.295		
**Type of therapy**					
Monotherapy	72 (59.5)	Referent		Referent	
Polytherapy	115 (35.0)	0.366 (0.238–0.561)	<0.001	0.367 (0.218–0.617)	<0.001

CI, confidence interval; Kg/m^2^, kilogram per square meter; OR, odds ratio; Rx, prescription.

* Old age (>55 years for males, ≥65 years for females).

The mean HbA1c% values of patients on visit 1 and 2 were, respectively 9.2 ± 1.2 and 8.8 ± 1.6. Based on definition used in the current study, a total of 156 (36.7%) and 178 (39.5%) patients, respectively had controlled glycemic levels on visit 1 and 2. In multivariate analysis, suffering from dyslipidemia (OR = 0.134, *p*-value = 0.001), CKD (OR=0.111, *p*-value = 0.030) and receiving sulfonylureas (OR = 0.156, *p*-value = 0.001) and guidelines compliant prescription (OR = 4.195, *p*-value = 0.003) had statistically significant associations with controlled glycemic level on visit 2. This model fit was based on non-significant Hosmer Lemeshow (*p*-value = 0.612) and overall percentage 84.4% from classification table (Table 5).

**Table 5:** Factors associated with glycemic control.

**Variables**	**Glycemic control No. (%)**	**Univariate analysis**		**Multivariate analysis**	
		**OR (95%CI)**	***p*** **-value**	**OR (95%CI)**	***p*** **-value**
**Gender**					
Male	125 (45.3)	Referent		Referent	
Female	53 (30.5)	0.529 (0.355–0.790)	0.002	0.565 (0.276–1.163)	0.121
Old age^*^					
No	162 (49.7)	Referent		Referent	
Yes	16 (12.9)	0.150 (0.085–0.215)	<0.001	0.481 (0.173–1.337)	0.161
**Body mass index** (kg/m^2^)					
Underweight (< 18.5)	5 (62.5)	Referent		Referent	
Normal (18.5–24.9)	59 (85.5)	3.540 (0.729–17.195)	0.117	1.135 (0.131–0.790)	0.909
Overweight (25.0–29.9)	93 (44.1)	0.473 (0.110–2.030)	0.314	0.414 (0.057–3.015)	0.384
Obese (>30.0)	21 (13.0)	0.089 (0.020–0.402)	0.002	0.446 (0.051–3.920)	0.467
**Comorbidities**					
No	128 (71.1)	Referent		Referent	
1–2	32 (42.1)	0.295 (0.169–0.516)	<0.001	0.706 (0.205–2.431)	0.581
>2	18 (9.3)	0.042 (0.023–0.074)	<0.001	0.274 (0.035–2.140)	0.217
**Hypertension**					
No	69 (72.6)	Referent		Referent	
Yes	109 (30.7)	0.167 (0.101–0.276)	<0.001	0.555 (0.227–1.360)	0.198
**Dyslipidemia**					
No	142 (69.3)	Referent		Referent	
Yes	36 (31.6)	0.076 (0.048–0.121)	<0.001	0.134 (0.040–0.454)	0.001
**Chronic kidney diseases**					
No	175 (48.1)	Referent		Referent	
Yes	3 (3.5)	0.039 (0.012–0.126)	<0.001	0.111 (0.015–0.805)	0.030
**Cardiovascular diseases**					
No	166 (49.4)	Referent		Referent	
Yes	12 (10.5)	0.120 (0.064–0.227)	<0.001	1.603 (0.377–6.812)	0.522
**Stroke**					
No	175 (43.6)	Referent		Referent	
Yes	3 (6.1)	0.084 (0.026–0.275)	<0.001	0.758 (0.166–3.465)	0.720
**Others**					
No	143 (42.1)	Referent		Referent	
Yes	35 (31.8)	2.165 (1.235–3.798)	0.007	1.401 (0.481–4.079)	0.536
**Metformin**					
No	10 (19.2)	Referent		Referent	0.730
Yes	168 (42.2)	3.068 (1.497–6.289)	0.002	1.304 (0.289–5.886)	
**Sulfonylurea**					
No	160 (53.3)	Referent		Referent	
Yes	18 (12.2)	0.119 (0.069–0.205)	<0.001	0.156 (0.052–0.566)	0.001
**Insulin**					
No	144 (72.5)	Referent		Referent	
Yes	34 (19.2)	0.213 (0.137–0.332)	<0.001	0.692 (0.305–1.569)	0.378
**SGLT-2 inhibitors**					
No	165 (43.9)	Referent		Referent	
Yes	13 (17.6)	0.213 (0.137–0.332)	<0.001	1.844 (0.506–0.6713)	0.354
**GLPRA**					
No	177 (40.1)	Referent		Referent	
Yes	1 (11.1)	0.186 (0.023–1.504)	0.115	3.390 (0.161–71.225)	0.432
**Gliptins**					
No	98 (40.5)	Referent		Referent	
Yes	80 (38.5)	0.918 (0.628–1.342)	0.660	(-)	
**Type of therapy**					
Monotherapy	75 (62.0)	Referent		Referent	
Polytherapy	103 (31.3)	0.280 (0.181–0.342)	<0.001	1.301 (0.431–3.922)	0.641
**Guidelines compliant Rx**					
No	66 (25.1)	Referent		Referent	
Yes	112 (59.9)	4.457 (2.976–6.676)	<0.001	4.195 (1.641–10.723)	0.003

CI, confidence interval; GLPRA, Glucagon-like Peptide-1 receptors agonists; Kg/m^2^, kilogram per square meter; OR, odds ratio; Rx, prescription; SGLT2, Sodium-glucose cotransporters-2;

* Old age (>55 years for males, ≥65 years for females).

## Discussion

Optimal glycemic control can significantly decrease the incidence of diabetic complications and enhance the patients' expectancy and quality of life. Pharmacotherapy in compliance with clinical practice guidelines contributes in achieving optimal control of diabetes. In current study, only 41.6% prescriptions were in compliance with ADA guidelines recommendations (7). As mentioned above, there is a scarcity of published studies which have evaluated doctors' objective adherence to pharmacotherapeutic recommendations of DM guidelines by using explicit criteria and reviewing patients' medical records, this made it difficult for us to compare our findings with previously published studies. However, similar percentage (39%) of guidelines compliant therapy modification in diabetic patients has been reported by a study conducted in the United States (15). On the other hand, a comparatively greater proportion (54.2%) of diabetic patients received guidelines' compliant prescriptions in Turkey (22).

Unless contraindicated, ADA guidelines recommend metformin as a first-line anti-diabetic (7). Therefore, the high rate of metformin prescription in present study (88.4%) was in line with guidelines recommendations and studies conducted elsewhere (37, 40). Moreover, its extensive availability, low cost and being a component of fixed dose combinations (FDC) could have also contributed to high prescription rate of metformin. In current cohort, Dipeptidyl peptidase-4 inhibitors commonly known as gliptins were the second most commonly prescribed anti-diabetics and were received by 44.6% patients. Guidelines recommend that if in a diabetic patient without comorbidity, first-line therapy is insufficient to control blood glucose levels, any one of gliptins, thiazolidinediones, sulfonylureas and SGLT2-I should be added as a second-line agent (7). Therefore, the relatively higher prescription rate of gliptins was compliant with guidelines recommendations and findings of studies conducted elsewhere (37, 41). The additional benefits of improved blood glucose control, cardio-protective effects, being weight neutral (42) and availability as FDC might be the other reasons for high prescription rates of gliptins.

In current cohort, patients who suffered from CKD and received multiple anti-diabetics were significantly less likely to receive guidelines compliant prescriptions. Presence of comorbidities complicates management of diabetes and has previously been reported as a risk factor for guidelines divergent prescriptions (36, 43). Upon cross-tabulation, we found that 85/86 diabetic CKD patients were treated with two or more anti-diabetics. Of these 85 patients, 45 were on two and 40 on three antidiabetics. As per ADA guidelines, metformin is contraindicated in CKD patients with eGFR < 30 ml/min/1.73 m^2^, and in CKD patients of stage III-a and III-b who are already on metformin, the maximum daily doses of metformin should be reduced to 2,000 mg and 1,000 mg, respectively (7). We found that 9/15 patients with end-stage renal disease received the contraindicated metformin, and its dose was not adjusted in 30 patients with CKD stage III. It was also observed that 23/45 diabetic CKD patients who received two anti-diabetics were not on guidelines recommended second line agents i.e., SGLT2i or GLP1-RA (7). The higher odds of guidelines divergent prescriptions in CKD patients and those who received polytherapy show that doctors deviated from guidelines while selecting the correct doses and second line agents in these patients. Moreover, as diabetes is often treated as a ‘glucose centric disease' (9), the incidence of adverse renal outcomes due to irrational doses and less preferred anti-diabetics might not have been a preference of the current cohort of doctors. Similar doctors' guidelines divergent practices among diabetic CKD patients have also been reported from Malaysia (23).

In current cohort, diabetes control was poor and only 39.5% patients were at goal glycemic level on visit 2. Likewise, in previously published studies glycemic control in diabetic patients ranged from 23.4 to 60% (3–6). In multivariate analysis, patients who suffered CKD, dyslipidemia and received sulfonylureas were significantly less likely to be at goal glycemic levels. Whereas, the odds of glycemic control was significantly higher in those who received guidelines compliant prescriptions. Due to metabolic acidosis, elevated levels of parathyroid hormone, and decreased level of vitamin D, early stages of CKD are associated with decreased production and resistance to insulin (44). As majority of diabetic CKD patients in current cohort had CKD stage-III, the significantly high prevalence of poor glycemic control in these patients is not astonishing. As glucose and lipid metabolism are interlinked to each other, many Type 2 diabetic patients usually suffer from dyslipidemia. Therefore, dyslipidemia is not only the consequence but also the cause of a disturbed glucose metabolism. It has been reported that elevated levels of triglycerides lead to elevated levels of free fatty acids which may induce insulin resistance and β-cell dysfunction, worsen glucose metabolism and make it more difficult to achieve glycemic goals in patients with hypertriglyceridemia (45). Similar finding of negative association between dyslipidemia and glycemic control have been reported by other studies conducted elsewhere (46, 47). In present study, patients who received sulfonylureas were significantly less likely to be at goal glycemic level. Sulfonylureas are potent hypoglycemic and reduce blood glucose levels by triggering insulin release from the pancreatic β-cells. However, by causing frequent episodes of hypoglycemia, progressive loss of effectiveness with chronic use and affecting cardiac potassium channels resulting in diminished response to ischemia, sulfonylureas are mostly used as third-line agents (48). Therefore, the current negative association between use of sulfonylureas and glycemic control could be due to their prescriptions to patients with severe diabetes. In current study, the likelihood of glycemic control was significantly greater in patients who received guidelines compliant prescriptions. Guidelines contain recommendations based on high quality evidence obtained from large and rigorously conducted randomized control trials, individual patients' data meta-analyses, systematic reviews and expert opinions. Therefore, guidelines compliant practices should eventually benefit patients. Similar positive association between guidelines compliant therapy and glycemic control has been reported by studies conducted elsewhere (18). In current study, in terms of writing guidelines adherent prescriptions, specialists and consultants performed better than medical officers. Likewise findings have been reported by studies conducted elsewhere (39, 49). Comparatively high qualification, greater familiarity with guidelines recommendations and being in practice for longer periods could be the possible reasons for this finding.

## Conclusion

Our findings demonstrate that both doctors' adherence to pharmacotherapeutic recommendations of ADA guidelines and glycemic control were poor. Doctors mostly diverted from guidelines while prescribing to diabetic CKD patients and selecting the second line anti-diabetic agents. The finding that guidelines compliant pharmacotherapy resulted in better glycemic control reflects that remedial steps should be taken to improve guidelines adherence particularly in patients who were at greater risk of receiving guidelines non-compliant prescriptions. Availability of clinical pharmacists as a full member of diabetes care team, computerized decision support tools based on guidelines recommendations, dissemination of guidelines and behavior modeling by opinion leaders may improve doctors' guideline adherence and patients' glycemic control.

### Limitations

This study has some notable limitations. Diabetic management is a multicomponent process involve screening, life-style modifications, pharmacological treatment and continued follow-up. However, the current study did not focus on overall diabetic care process but only evaluated doctors' anti-diabetic prescribing practices. This study also lacks information about patients' medication adherence. It did not evaluate the pharmacotherapeutic management of comorbidities like hypertension, dyslipidemia, CVD etc., and its impact on patients' glycemic levels. Furthermore, DM is a chronic condition and requires a long observation period to decide about its control. Even though, to ensure that HbA1c level noted on final visit was the representative glycemic level of patients, we enrolled only established diabetic patients and excluded those whom follow-up visits were schedule in a span of <3 months.

## Data availability statement

The raw data supporting the conclusions of this article will be made available by the authors, without undue reservation.

## Ethics statement

The studies involving human participants were reviewed and approved by Ethical Institutional Review Board (IRB), Faculty of Pharmacy and Health Sciences, University of Balochistan, Quetta. The patients/participants provided their written informed consent to participate in this study.

## Author contributions

TS, AW, and QI conceptualized the study. TS and AK collected the data. YMA and MNI analyzed the data. NA and MA wrote the manuscript. NA also supervised the study. All the authors contributed to the article and approved the submitted version.

## Conflict of interest

The authors declare that the research was conducted in the absence of any commercial or financial relationships that could be construed as a potential conflict of interest.

## Publisher's note

All claims expressed in this article are solely those of the authors and do not necessarily represent those of their affiliated organizations, or those of the publisher, the editors and the reviewers. Any product that may be evaluated in this article, or claim that may be made by its manufacturer, is not guaranteed or endorsed by the publisher.
