# Supplementary material for: Doctors' adherence to guidelines recommendations and glycaemic control in diabetic patients in Quetta, Pakistan: Findings from an observational study
Source: Front Med (Lausanne). 2022 Oct 31;9:978345. doi: 10.3389/fmed.2022.978345 (PMC9661729; doi:10.3389/fmed.2022.978345)
Supplement: Supplementary file 1 [file Data_Sheet_1.pdf]

Criteria for Measuring Adherence to ADA (2019) Guidelines (Supplementary Chart 1)

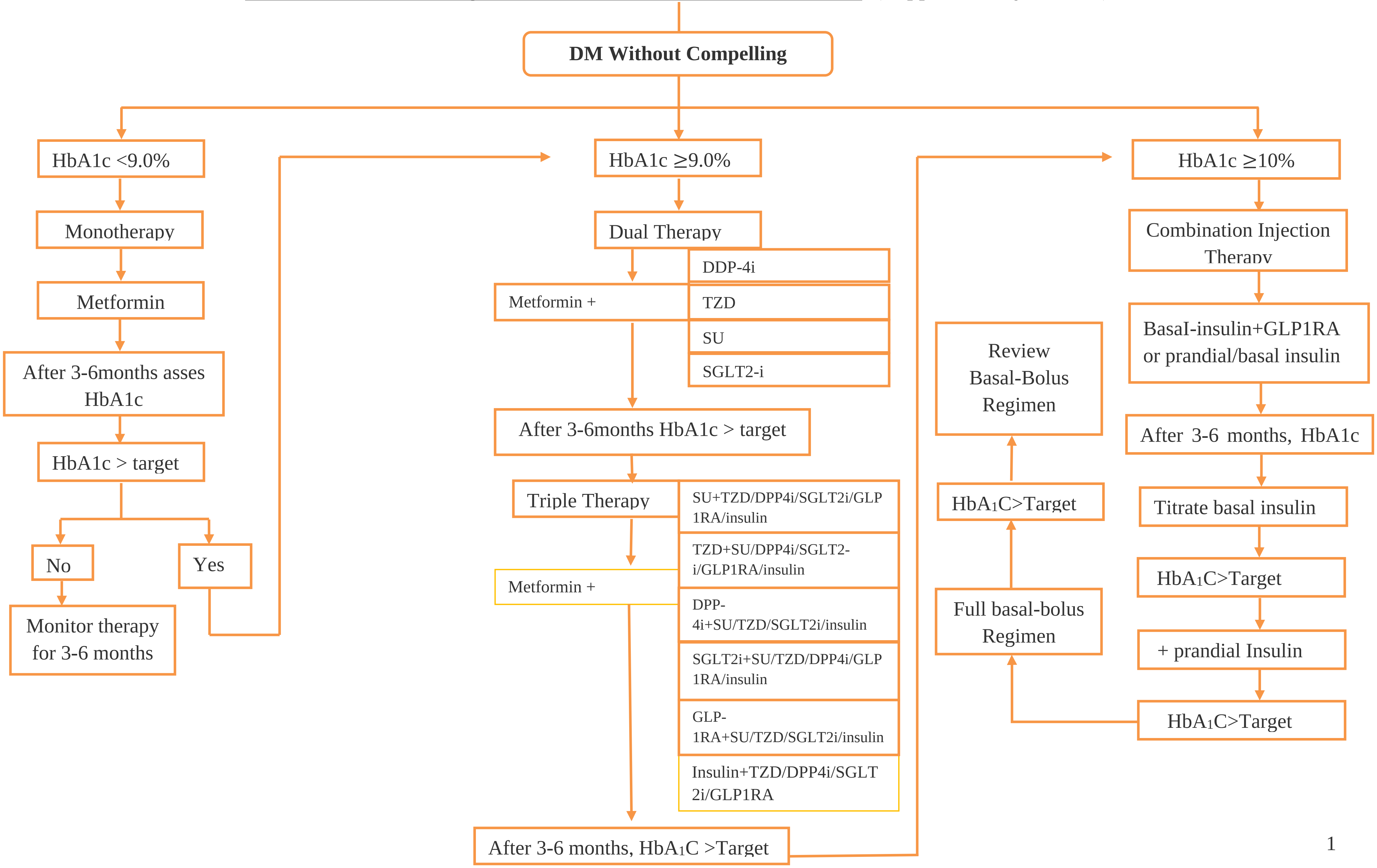

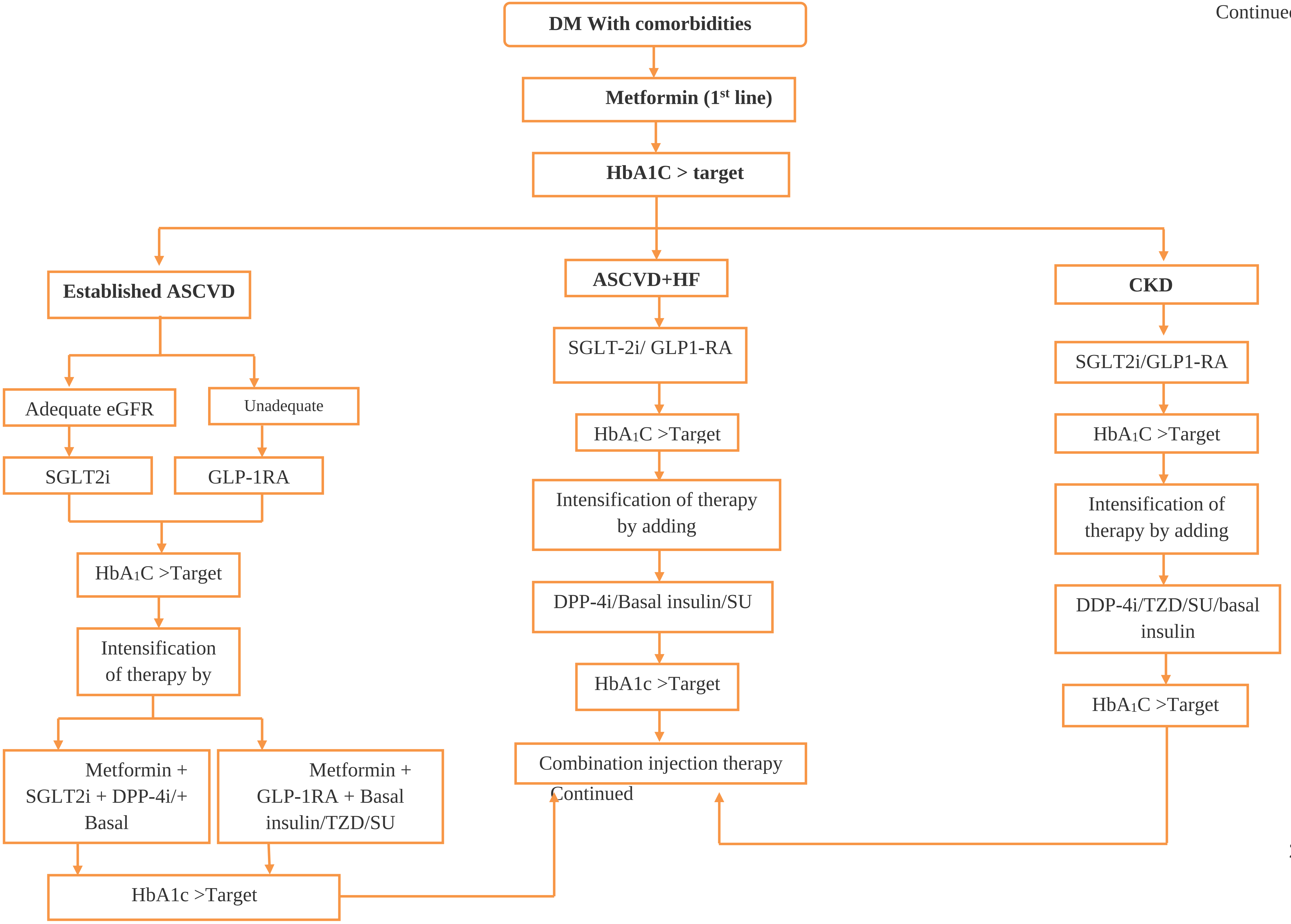
DM With comorbidities
Metformin (1st line)
HbA1C > target
Established ASCVD
ASCVD+HF
CKD
Adequate eGFR
Unadequate
SGLT2i
GLP-1RA
HbA1C >Target
Intensification of therapy by
Metformin + SGLT2i + DPP-4i/+ Basal
Metformin + GLP-1RA + Basal insulin/TZD/SU
HbA1c >Target
SGLT-2i/ GLP1-RA
HbA1C >Target
Intensification of therapy by adding
DPP-4i/Basal insulin/SU
HbA1c >Target
Combination injection therapy
Continued
SGLT2i/GLP1-RA
HbA1C >Target
Intensification of therapy by adding
DDP-4i/TZD/SU/basal insulin
HbA1C >Target

Continued

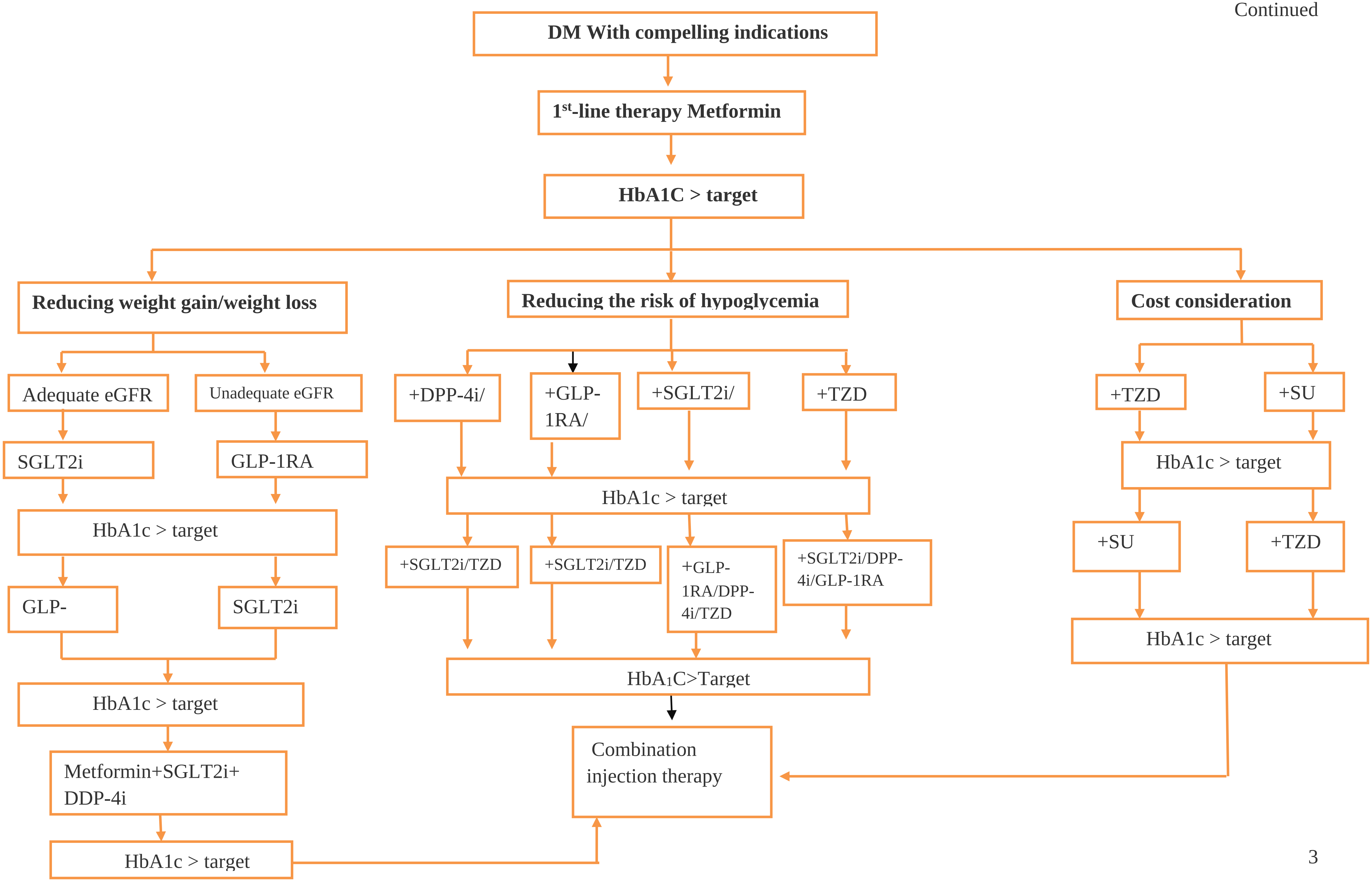
DM With compelling indications
1st-line therapy Metformin
HbA1C > target
Reducing weight gain/weight loss
Reducing the risk of hypoglycemia
Cost consideration
Adequate eGFR
Unadequate eGFR
SGLT2i
GLP-1RA
HbA1c > target
GLP-
SGLT2i
HbA1c > target
Metformin+SGLT2i+ DDP-4i
HbA1c > target
+DPP-4i/
+GLP-1RA/
+SGLT2i/
+TZD
HbA1c > target
+SGLT2i/TZD
+SGLT2i/TZD
+GLP-1RA/DPP-4i/TZD
+SGLT2i/DPP-4i/GLP-1RA
HbA$_1$C>Target
Combination injection therapy
+TZD
+SU
HbA1c > target
+SU
+TZD
HbA1c > target

HbA1c: Hemoglobin A1c; ADA: American diabetes association; DPP-4: Dipeptidyl peptidase-4; TZD: Thiazolidine; SU: Sulfonylurea; SGLT2i: Sodium/glucose cotransporter-2 inhibitors; GLP-1 RA: glucagon-like peptide 1 Receptor agonists; ASCVD: Atherosclerotic cardiovascular disease; HF: Heart failure; CKD: Chronic kidney disease

Figure 2.2 criteria for measuring doctors' adherence to ADA 2019 guideline recommendation
